# Urinary obstruction caused by urethral stones in a pediatric patient with hypospadias: a rare Case Report in Somalia

**DOI:** 10.1016/j.eucr.2025.103323

**Published:** 2025-12-23

**Authors:** Amal Abdullahi Abdi, Abdullahi Hassan Elmi, Fahmo Hussein Ibrahim

**Affiliations:** a Department of Nursing and Midwifery, Dr. Sumait Hospital, Faculty of Medicine and Health Sciences, SIMAD University, Mogadishu, Somalia; b Department of Obstetrics and Gynecology, Dr. Sumait Hospital, Faculty of Medicine and Health Sciences, SIMAD University, Mogadishu, Somalia

**Keywords:** Urethral stones, Pediatric urinary retention, Hypospadias, Urinary obstruction, Hypospadias repair

## Abstract

Urethral stones are an uncommon cause of urinary blockage in children, particularly in those with congenital anomalies such as hypospadias. We present a 5-year-old boy with distal hypospadias who experienced repeated episodes of urinary retention and intermittent painful urination. During surgical repair, small urethral stones were identified at the stenotic meatus. The patient underwent a successful tubularized-incised plate hypospadias repair, resulting in normal voiding and resolution of obstruction. This case highlights the importance of considering urethral calculi in children with congenital urethral anomalies to prevent recurrent urinary retention and related complications.

## Highlights

• Urethral calculi are a rare but important cause of urinary obstruction in children.
• Congenital anomalies like hypospadias can predispose to urinary stasis and stone formation.
• Small distal urethral stones may be missed on imaging, requiring careful clinical evaluation.
• Surgical repair of hypospadias can resolve obstruction and prevent recurrence.
• Early recognition and intervention improve outcomes and preserve urinary function.

## 1 Introduction

Urethral stones are a rare cause of urinary blockage in children and are even less common in those with congenital conditions like hypospadias. We describe a young boy with hypospadias who had repeated episodes of difficulty urinating and intermittent painful urination. Investigation revealed small stones in his urethra as the underlying cause. This case emphasizes the need for clinicians to consider urethral calculi in children with congenital urethral anomalies who present with urinary obstruction.

## 2 Case report

A 5-year-old boy was brought to the emergency department with a 14-h history of urinary difficulty. His parents reported that he could only pass urine in small drops, without associated pain. They also observed solid, white material at the urethral opening. During transport to the hospital, the child was able to urinate spontaneously. The family noted three previous similar episodes over the past year, all of which resolved on their own.

On examination, the child appeared well and in no distress. Genitourinary assessment revealed hypospadias with a partially dorsal-hooded foreskin and a small, stenotic sub-coronal meatus. No meatal inflammation or stones were observed at the time of evaluation. The scrotum appeared normal, with both testes descended bilaterally.

Family history was notable for distal hypospadias in the patient's father, who had recently been diagnosed with a varicocele. Urinalysis revealed positive nitrites and leukocyte esterase, with a urine pH of 6.4. Microscopic examination showed over 61 white blood cells, 4–6 red blood cells, and trace amorphous crystals. Urine culture subsequently grew more than 100,000 colony-forming units of coagulase-negative *Staphylococcus species*. Laboratory evaluation showed a white blood cell count of 14.8 K/μL and hemoglobin of 11.76 g/dL, while serum creatinine was 0.41 mg/dL.

A retroperitoneal ultrasound demonstrated kidneys of normal appearance and a thick-walled bladder with layering debris. No calculi were identified in the kidneys or bladder. Post-void residual volume was not assessed.

At presentation, the patient's urinary retention was initially attributed to meatal stenosis. He was discharged on a four-day course of cephalexin at 50 mg/kg. The patient later attended an outpatient follow-up for further evaluation of his hypospadias and meatal stenosis. After discussion with the family, a decision was made to proceed with surgical repair of the hypospadias.

**Fig. 1:**
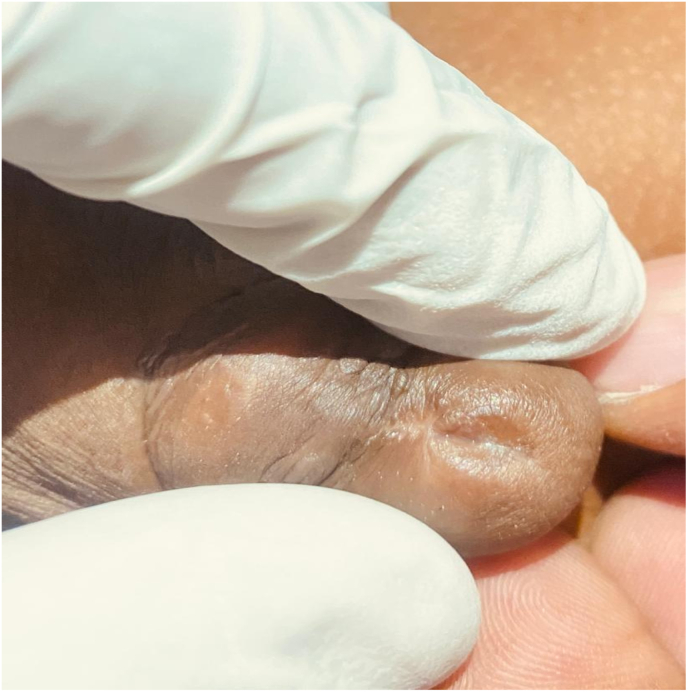
A tiny calculus identified at the constricted urethral opening.

On the day of surgery, five weeks later, the patient's mother reported that he had experienced multiple additional episodes of urinary difficulty. She described manually expressing small stones from his urethra to facilitate urination. During the operation, a small stone was identified at the stenotic urethral meatus (Fig. 1).

Due to the small size of the stone and local resource limitations, stone composition analysis was not performed. No additional stones or foreign bodies were identified intraoperatively. Formal endoscopic evaluation of the proximal urethra and bladder was considered but not performed, as no proximal obstruction or intraluminal pathology was suspected based on operative findings.

Surgical repair was carried out using a tabularized-incised plate technique, with a 5 Fr feeding tube placed as a urethral catheter. On postoperative day 1, the patient presented urgently to the clinic with abdominal pain and peri-catheter urine leakage, which was resolved after flushing and aspirating the catheter. No calculi were identified as the cause of obstruction. The catheter was removed on postoperative day 11. At follow-up, the urethral meatus was orthotopic and of normal caliber. The patient was voiding normally, and the parents reported no further episodes of urinary retention or urethral stones.

## 3 Discussion

Urethral calculi are an uncommon cause of urinary obstruction in children, representing less than 1 % of pediatric urinary stones.^1^^,^^2^ Their occurrence in patients with congenital anomalies such as hypospadias is particularly rare, as anatomical irregularities can promote urinary stasis and predispose to stone formation.^3^, ^4^, ^5^ In the present case, a 5-year-old boy with distal hypospadias experienced recurrent urinary retention and intermittent painful urination, ultimately found to have small urethral stones contributing to his symptoms.

The development of urethral calculi in children is typically multifactorial, involving urinary stasis, infection, and anatomical obstruction.^6^^,^^7^ In this patient, the stenotic sub-coronal meatus likely impeded normal urine flow, creating conditions favorable for stone formation. Additionally, the documented urinary tract infection with coagulase-negative *Staphylococcus species* may have promoted crystallization and aggregation of urinary sediments. While imaging such as retroperitoneal ultrasound is useful in evaluating the upper urinary tract and bladder, distal urethral stones are often missed, underscoring the importance of clinical suspicion and careful physical examination.^2^^,^^8^

Management of pediatric urethral calculi depends on stone size, location, and underlying anatomical abnormalities. Small distal stones may be manually expressed, as observed in this patient, whereas impacted or proximal stones frequently require endoscopic or surgical removal.^3^ In children with hypospadias, surgical repair not only corrects the anatomical defect but also reduces the risk of recurrent obstruction and stone formation. The tubularized-incised plate repair performed in this case led to normalization of voiding and complete resolution of urinary retention, demonstrating the dual benefit of addressing both the structural anomaly and the obstructive complication.^6^^,^^8^

This case has several limitations. Stone composition analysis was not available, limiting definitive conclusions regarding stone etiology. Post-void residual volume and formal assessment of voiding frequency and bladder emptying were not documented. In addition, cystoscopic evaluation of the proximal urethra and bladder was not performed; therefore, occult pathology cannot be entirely excluded. Nevertheless, the clinical course and postoperative outcome support meatal obstruction as the primary contributor to stone formation.

This case highlights the need for clinicians to maintain a high index of suspicion for urethral calculi in pediatric patients with congenital urethral anomalies who present with recurrent urinary obstruction. Early recognition and timely intervention can prevent persistent urinary retention, infection, and potential long-term complications affecting the lower urinary tract. Postoperative monitoring is also crucial to ensure catheter function and detect any early signs of obstruction or infection. although urethral calculi are rare in children, they should be considered in those with hypospadias and recurrent urinary retention. A combination of careful clinical evaluation, appropriate imaging, and timely surgical intervention can result in excellent outcomes, as demonstrated in this patient from Somalia.

## 4 Conclusion

Although rare, urethral stones should be considered in children with hypospadias who present with recurrent urinary retention. Early recognition and timely surgical management not only relieve obstruction but also correct underlying anatomical anomalies, ensuring long-term normal urinary function and preventing recurrence.

## CRediT authorship contribution statement

**Amal Abdullahi Abdi:** Writing – original draft, Conceptualization. **Abdullahi Hassan Elmi:** Writing – review & editing, Writing – original draft, Conceptualization. **Fahmo Hussein Ibrahim:** Supervision.

## Ethics and consent

Written consent for publication, including the use of clinical information and images, was obtained from the patient's parent, as the patient is under 18 years of age. Ethical approval from the institution was not required according to local guidelines for single case reports.

## Funding

The authors did not receive any funding for the research, preparation, or publication of this study.

## Conflicts of interest

The author reports no conflicts of interest associated with this study.
